# Long-Term Prediction of the Demand of Colonoscopies Generated by a Population-Based Colorectal Cancer Screening Program

**DOI:** 10.1371/journal.pone.0164666

**Published:** 2016-10-12

**Authors:** Mercè Comas, Joan Mendivil, Montserrat Andreu, Cristina Hernández, Xavier Castells

**Affiliations:** 1 Epidemiology and Evaluation Department, Hospital del Mar, Barcelona, Spain; 2 IMIM (Hospital del Mar Medical Research Institute), Barcelona, Spain; 3 Red de Investigación en Servicios de Salud en Enfermedades Crónicas (REDISSEC), Barcelona, Spain; 4 CPS Market Access, Roche Diagnostics International Ltd., Rotkreuz, Switzerland; 5 Gastroenterology Department, Hospital del Mar, Barcelona, Spain; Institut d'Investigacions Biomèdiques August Pi i Sunyer (IDIBAPS), SPAIN

## Abstract

**Objective:**

To estimate the long-term need for colonoscopies after a positive fecal immunochemical test (FIT) and post-polypectomy surveillance in the context of a population-based colorectal cancer (CRC) screening program.

**Methods:**

A discrete-event simulation model was built to reproduce the process of CRC screening and post-polypectomy surveillance following European guidelines in a population of 100,000 men and women aged 50–69 years over a 20-year period. Screening consisted of biennial FIT and colonoscopy in participants with positive results. The model was mainly fed using data from the first and second rounds of a Spanish program (2010–2013). Data on post-polypectomy surveillance results were obtained from the literature. A probabilistic multivariate sensitivity analysis was performed on the effect of participation, FIT positivity, and adherence to surveillance colonoscopies. The main outcome variables were the number of colonoscopies after a positive FIT, surveillance colonoscopies, and the overall number of colonoscopies.

**Results:**

An average yearly number of 1,200 colonoscopies after a positive FIT were predicted per 100,000 inhabitants with a slight increase to 1,400 at the end of the 20-year period. Surveillance colonoscopies increased to an average of 1,000 per 100,000 inhabitants in the long-term, showing certain stabilization in the last years of the 20-year simulation horizon. The results were highly sensitive to FIT positivity.

**Conclusions:**

Implementing a population-based CRC screening program will increase the demand for colonoscopies, which is expected to double in 20 years, mainly due to an increase in surveillance colonoscopies.

## Introduction

Colorectal cancer (CRC) has the highest incidence and the second highest mortality among cancers in Europe in both genders, with 446,000 new cases/year and 214,000 deaths/year.[1] Estimates from multiple randomized clinical trials show that CRC screening reduces CRC mortality by 15% to 33% and that this reduction holds steady when participants are followed-up for 30 years.[2]

The European Guidelines for Quality Assurance in Colorectal Cancer Screening and Diagnosis (the guidelines onwards)[3] set high standards for the continuum of population-based CRC screening programs. These guidelines not only address the diagnostic process but also provide a framework that includes invitation, organization, diagnosis, and the management of detected lesions. The document provides no recommendation for any particular screening strategy over the others based on cost-effectiveness, and the screening interval and age of the target population vary according to the best available evidence at the time of publication. Currently, most European population-based programs use biennial fecal occult blood tests with a colonoscopy in participants with a positive result.[4] Fecal immunochemical tests for hemoglobin (FIT) have been shown to be superior to guaiac fecal occult blood tests (gFOBT) in terms of increased participation and detection rates, both for adenomas and cancer,[5–7] and are currently becoming the preferred option throughout Europe.[4] However, the use of FIT directly impacts the demand for resources because its better outcomes depend on an expanding colonoscopy capacity.[8,9]

In addition, the guideline recommendation with possibly the strongest impact on health services is that for surveillance following adenoma removal. Based on the number and size of the adenomas, patients can be divided into risk groups with respect to their risk of developing advanced adenomas and cancer, and follow-up recommendations depend on the risk group. The adoption of these recommendations may put pressure on endoscopy services but deviations from them can increase demand even more, as recently shown in the context of an Italian screening program.[10]

A determined action toward the implementation of a program with these strict quality characteristics at the population level requires an accurate forecast of the resources involved. Forecasting is paramount, since a CRC screening program generates demand for health services (i.e. surveillance colonoscopies) over many years and has implications ranging from medical education to capacity allocation. To a certain extent, the increased demand may be offset by a reduced demand for diagnostic colonoscopy services, although population aging will further influence changes in demand.[11] In addition, screening-related endoscopy supply requires accurate planning of medical manpower because the technical expertise needed to meet the quality standards requires several years of clinical training.

Patient acceptance, screening compliance, capital versus operational costing, and the colonoscopy and human resources required are key determinants of the decisions taken by policy-makers and health services planners.[12] It is therefore crucial to assess the impact of each of these determinants by proposing different scenarios. Modelling methods are a powerful tool for this type of assessment[13,14] and can be applied to provide information on key capacity determinants of the short-term feasibility and the long-term sustainability of population-based CRC screening programs. Some studies have evaluated the actual costs of the first and repeat CRC screening rounds[15,16], while others have modelled the resource requirements and health outcomes of different CRC screening programs.[8,17,18]

Since there have been no studies on the long-term resources required to attain the post-polypectomy surveillance recommended by the European Guidelines in the context of a population-based CRC screening program, we aimed to estimate the number of colonoscopies derived from such a program in a population of 100,000 people over a 20-year period.

## Materials and Methods

### Ethics Statement

This study was approved by the ethics committee of Hospital del Mar. The need for written informed consent was waived because all data were analyzed anonymously.

### Discrete-event simulation model

**Fig 1:**
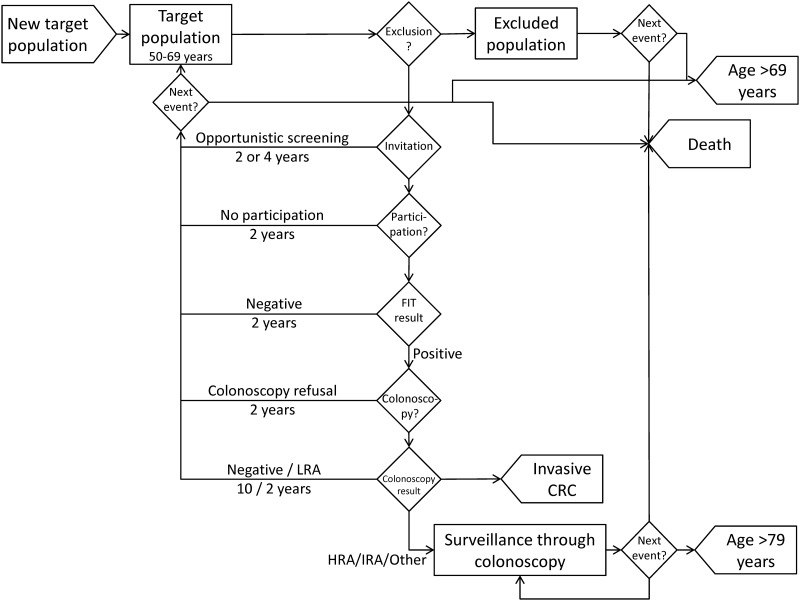
Conceptual model. CRC: Colorectal Cancer; HRA: High Risk Adenomas; IRA: Intermediate risk adenomas; LRA: Low Risk Adenomas.

A discrete-event simulation model[13] was built to reproduce the process of men and women entering a population-based CRC screening program. The real-world program invites women and men aged 50–69 years to biennial screening through FIT with colonoscopy for positive results. The population may be excluded from the screening program before invitation or when a CRC or adenoma is detected. Surveillance of detected adenomas was included in the model. The events simulated (Fig 1) were as follows: inclusion of a new person in the target population, exclusion process, invitation process, participation process, FIT result, colonoscopy after a positive FIT and surveillance colonoscopy. Detection of invasive CRC, death and exclusion from the target population due to age over 69 years were exits from the model. Individuals under colonoscopy surveillance had an age limit of 80 years. Individuals were invited to the program after 2 years in the following cases: no participation, participation with a negative FIT, colonoscopy refusal after a positive FIT, findings of low-risk adenomas or opportunistic screening through colonoscopy 5 years previously. Individuals were invited after 4 years if they had a colonoscopy (opportunistic screening) 3 years previously, while those with a negative result of colonoscopy after a positive FIT were invited after 10 years to routine screening.

A simulated time horizon of 20 years (from 2015 to 2034) was chosen to encompass the life history of a person entering a screening program (from 50 to 69 years) and to allow long-term prediction of colonoscopy demand according to the program results. Individual persons were simulated. All persons underwent biennial screening from 50 to 69 years. Persons aged 70 years or older were followed-up until 79 years only if they were undergoing surveillance colonoscopies. The study included the entire population involved in the system each year. Thus, individual people entering and exiting the model were simulated throughout the simulation horizon.

The simulation model was implemented by using Arena (Rockwell Software) version 14.5.

### Target population

The target population at the beginning of the simulation included 100,000 men and women aged 50–69 years undergoing biennial screening. From the second year on, persons aged 50 years old entered the target population every year, following current predictions on the Spanish population.[19] Persons aged 68–69 years were excluded from the target population after their last screening round. Age was assigned according to gender and time to death was assigned according to age and gender.

### Screening events

Data from the first and second rounds of a Spanish CRC Screening Program including 31 Basic Healthcare Areas was used to calculate the screening-related parameters (see S1 File Supporting Information for details). The percentage of exclusions, opportunistic screening, participation, positivity and colonoscopy refusal were treated as probabilistic parameters (see S1 File Supporting Information for details). As they were found to differ significantly by age groups and gender, different distributions were estimated for each parameter and for strata combining gender and the age groups 50–54 years, 55–59 years, 60–64 years and 65–69 years.

Exclusions from the target population before invitation were taken into account. Exclusions due to medical reasons included a personal history of CRC, adenomas or inflammatory bowel disease, or a familial history of CRC. Due to significant differences, the percentage of exclusions was calculated for initial screening by age groups and gender. For successive screening, the percentage of exclusions was based solely on exclusions for medical reasons (see S1 File Supporting Information for details).

The invitation process included opportunistic screening, which represents the invited population reporting they had had a colonoscopy within the previous 5 years. Those individuals assigned to opportunistic screening were not excluded from the target population but were invited after one or two rounds (2 or 4 years), depending on the time since the last colonoscopy (see S1 File Supporting Information).

The participation process included two stages. The first represented the pick-up of the FIT at the pharmacy. Persons returning the test were considered as participants, while those not picking-up or not returning the test were considered as non-participants and returned to the target population to be invited after 2 years. The probability of participation differed according to initial or successive screening: for the former, the probability was sampled by age group and gender and for the latter, it differed according to participation behavior in the previous round (see S1 File Supporting Information).

The result of FIT could be negative (less than 100 ng of hemoglobin per mL), in which case individuals returned to the target population and were invited after 2 years. The positivity of the FIT test differed by screening number, age and gender, thus, it was sampled according to age groups and gender for initial screening and a different distribution was used for successive screening (see S1 File Supporting Information). A colonoscopy was offered to patients with a positive FIT result.

Colonoscopy refusal was sampled by age group and gender and was treated as a probabilistic parameter (see S1 File Supporting Information). Individuals assigned to refuse the colonoscopy returned to the target population to be invited 2 years later.

Second-look colonoscopies (within 1 year) could be indicated in some cases (inappropriate preparation, additional polypectomy or follow-up of resection completeness). A value of 12.3%[20] was included in the model.

**Fig 2:**
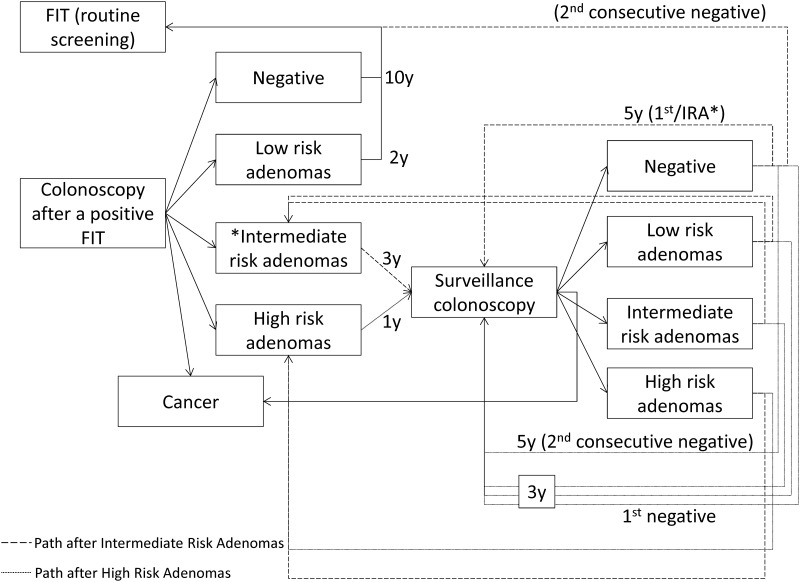
Conceptual model for surveillance after findings in the colonoscopy after a positive FIT. FIT: Fecal-occult Immunochemical Test; y: years.

Colonoscopy results were classified according to the European guidelines[3] in the following groups: normal, low-risk adenomas, intermediate-risk adenomas, high-risk adenomas and invasive cancer. The classification and distribution of results by initial and successive screening is depicted in S1 File Supporting Information. For intermediate- and high-risk adenomas, the follow-up scheme through surveillance colonoscopies also followed the recommendations of the European guidelines[3] and is depicted conceptually in Fig 2. The model assumed that surveillance colonoscopies would be carried out until the age of 79 years and that adherence would be 100% for the first surveillance colonoscopy and between 20% and 90% for successive surveillance colonoscopies (see S1 File Supporting Information).

### Probabilistic sensitivity analysis

Crucial parameters such as positivity, participation, exclusions due to personal history, opportunistic screening and colonoscopy refusal were included as probabilistic parameters at the individual level (see S1 File Supporting Information for details on the distributions assigned). Adherence to surveillance colonoscopies was also a probabilistic parameter but was constant for each run.

### Simulation analysis of results

The time units were years and the simulation horizon was 20 years, from 2015 to 2034. A total of 1,750 replications of the model with independent streams of random numbers were run. This sample size allowed the results to be stratified for the sensitivity analysis. Mean participation, positivity and adherence to surveillance colonoscopies by year and run were used for the sensitivity analysis. For validation purposes, a subgroup of runs representing the current scenario was analyzed. This group included 395 runs and was defined as a mean 20-year participation between 40% and 60%, a mean 20-year positivity between 4.7% and 6.8% and adherence to surveillance colonoscopies between 35% and 70%. This group of 395 runs was used for the main analysis of this study.

Results considered as definitive were checked by the research team by comparing them with real data from the first and second rounds of a Spanish screening program and the model was considered as valid, credible and useful for the purposes of the study (see S1 File Supporting Information).

Finally, the sensitivity analysis was run on the 1,750 runs to assess the impact of increasing participation, FIT positivity and adherence to surveillance colonoscopies by 1% on the number of colonoscopies (after a positive FIT, surveillance and overall colonoscopies). The importance and magnitude of each parameter were assessed through multivariate linear regression models including the interactions of all parameters with time and the interaction of positivity and participation.

The funding sources had no role in designing the study, interpreting the data, writing, or publishing the report. Further information about the data and methods used should be addressed to the corresponding author.

## Results

### Current scenario

Biennial screening of an initial population of 100,000 men and women led to a mean of around 50,000 invitations to the program per year, with an increase through time in both in the number of invitations and the number and percentage of participants because of population aging and the increase in successive screenings (Table 1). This had an impact on the number of colonoscopies after a positive FIT result (ranging from 1,218 in 2015 to 1,426 in 2034), although positivity, as a percentage, decreased through time (from 6.7% in 2015 to 4.9% in 2034), also because of the increase in successive screenings. The number of surveillance colonoscopies, with a mean adherence of 52.1%, began to increase from 2016 onwards and sharply increased in the long term, reaching 1,011 surveillance colonoscopies in 2034 (95% confidence interval from 974 to 1,029).

**Table 1:** Average yearly results in the short-, mid- and long-term (n = 395 simulations).

	Yearly results											
	2015			2020			2024			2034		
	N	95%CI	%	N	95%CI	%	N	95%CI	%	N	95%CI	%
Invited population	44,573	[44,518;44,600]		48,240	[48,150;48,285]		51,531	[51,420;51,586]		58,555	[58,438;58,613]	
Participants	19,513	[19,257;19,642]	43.8%	24,002	[23,568;24,223]	49.8%	26,308	[25,822;26,555]	51.1%	30,407	[29,837;30,697]	51.9%
Successive	0		0.0%	16,569	[16,197;16,758]	69.0%	19,899	[19,437;20,134]	75.6%	24,604	[24,058;24,882]	80.9%
Positive results of FIT	1,299	[1,261.1;1,318.8]	6.7%	1,250	[1,206.9;1,272.1]	5.2%	1,327	[1,283.5;1,349.8]	5.0%	1,496	[1,449.3;1,519.5]	4.9%
Number of colonoscopies after a positive FIT^a^	1,218	[1,180.4;1,237.8]	82.2%	1,193	[1,150.0;1,215]	83.7%	1,257	[1,212.2;1,279.7]	83.0%	1,426	[1,379.7;1,449.5]	83.6%
Negative	346	[329.1;354.7]	32.0%	405	[384.8;414.6]	38.2%	441	[420.5;451.6]	39.3%	507	[484.3;519.1]	40.0%
Positive no cancer	667	[642.2;680.0]	61.7%	609	[582.9;622.2]	57.5%	638	[610.7;651.7]	56.9%	714	[686.8;727.5]	56.2%
Low-risk adenomas	189	[177.4;195.0]	17.5%	204	[190.6;210.5]	19.2%	215	[201.8;222.5]	19.2%	252	[236.7;259.2]	19.8%
Intermediate-risk adenomas	297	[281.5;305.1]	27.5%	284	[267.9;292.3]	26.8%	296	[279.6;304.9]	26.4%	328	[310.6;336.5]	25.8%
High-risk adenomas	181	[168.2;187.6]	16.7%	121	[111.0;126.3]	11.4%	126	[115.6;131.3]	11.2%	134	[125.0;139.2]	10.6%
Positive for cancer	68	[61.0;71.8]	6.3%	46	[40.5;49.0]	4.4%	43	[37.3;45.9]	3.8%	48	[42.3;51.0]	3.8%
Results of surveillance colonoscopy^b^				546	[524.5;557.3]		873	[842.0;888.1]		1,011	[974.1;1,029.3]	
Negative				391	[373.0;399.7]	71.5%	621	[595.8;633.6]	71.2%	719	[690.3;733.2]	71.1%
Positive no cancer				154	[142.7;160.1]	28.2%	249	[234.9;255.9]	28.5%	288	[272.0;296.2]	28.5%
Low-risk adenomas				141	[129.6;146.2]	25.7%	226	[212.6;232.5]	25.9%	260	[244.7;267.8]	25.7%
High- and Intermediate-risk adenomas				14	[10.3;15.3]	2.5%	23	[19.1;25.1]	2.6%	28	[23.4;30.4]	2.8%
Positive for cancer				1	[0.4;1.9]	0.3%	3	[1.4;3.6]	0.3%	4	[2.1;4.8]	0.4%

^a^Number includes repeated colonoscopies, the % was calculated for the first colonoscopy.

^b^Average adherence to surveillance colonoscopies: 52.1%.

FIT: Fecal-occult immunochemical test. CI: Confidence Interval

**Fig 3:**
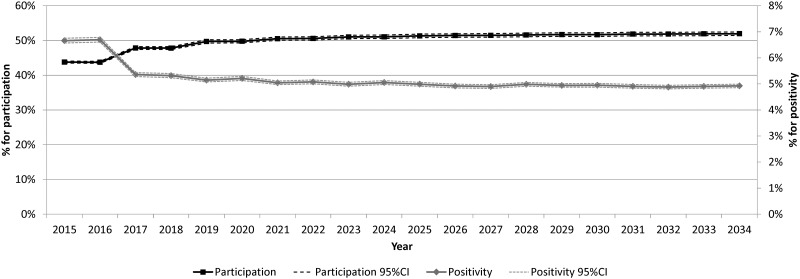
Percentage of participation and positivity of FIT, by year (n = 395 runs). FIT: Fecal-occult Immunochemical Test. CI: Confidence Interval.

Variations in mean participation and mean positivity through time are shown in Fig 3. The first 2 years represent the first round of the program and show higher positivity and lower participation because all participants underwent initial screening. Afterward, the greater number of successive screenings (80.9% in 2034) impacted on an increasing participation rate (from 43.8% in 2015 to 51.9% in 2034) and a decreasing positivity, both stabilizing in the long-term.

**Fig 4:**
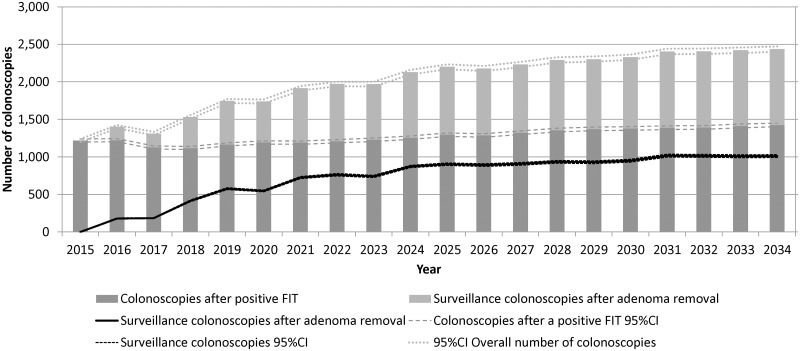
Number of colonoscopies after a positive FIT and surveillance colonoscopies, by year (n = 395 runs). FIT: Fecal-occult Immunological Test. CI: Confidence Interval.

The main outcome of this study was the number of colonoscopies needed for both the screening program and for surveillance of non-cancer findings. Fig 4 shows the number of colonoscopies in stacked bars: below, those after a positive FIT, which slightly increased over time, above, the number of surveillance colonoscopies. The number of surveillance colonoscopies is repeated as a line to show its sharp increase during the first 10 years and stabilization in the last years. The overall number of colonoscopies doubled after 20 years (from 1,218 to 2,437).

### Probabilistic sensitivity analysis

**Fig 5:**
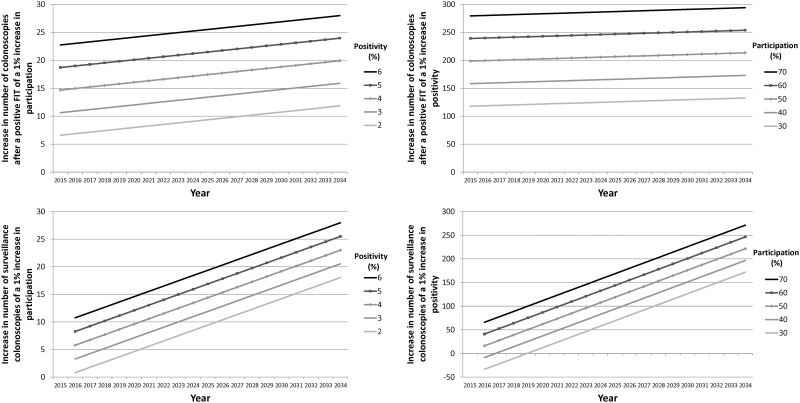
Magnitude of the effects of participation and positivity, by year and type of colonoscopy. Left panel: impact, by year, of a 1% increase in participation on the number of colonoscopies according to different positivity values. Right panel: impact, by year, of a 1% increase in positivity on the number of colonoscopies according to different values of participation.

The results of the 1,750 runs were used for a multivariate sensitivity analysis (see S1 File Supporting Information for details). The statistical significance of interactions raised the need to interpret the effects of participation, positivity and time jointly. Fig 5 represents the magnitude of the effects of participation and FIT positivity over time on the number of colonoscopies (after a positive FIT and surveillance). The lines in the left panel show the increase due to a 1% increase in participation, by year, in the number of colonoscopies according to different positivity values (from 2% to 6%). The lines in the right panel show the increase due to a 1% increase in positivity, by year, in the number of colonoscopies according to different participation values (from 30% to 70%). A 1% increase in positivity had an impact comparable to that of a 10% increase in participation at the end of the 20-year period, the highest impact represented being an increase of 294 colonoscopies after a positive FIT for a participation of 70% in the year 2034. Adherence had a lower impact than the rest of the parameters, although its magnitude also increased over time. The impact of a 1% increase in adherence to surveillance colonoscopies was not statistically significant until 2019, with an increase of 1 colonoscopy, which increased to 6 colonoscopies in 2034.

## Discussion

Our study presents a discrete-event simulation model of a population-based colorectal screening program that complies with the European Guidelines and provides a valuable source of information for healthcare services planning, as it predicts the future demand for colonoscopies, not only those following positive screening tests, but also those colonoscopies needed to follow the recommended surveillance of patients with intermediate- or high- risk adenomas.

Previous research on the demand and capacity for colonoscopy in the United States raised a red flag that prompted close evaluation of these two factors during the planning phase of CRC screening programs. In Europe, interest in this topic is equally high, given the directives from both political and scientific parties regarding the population-based approach to CRC screening. The upside of this approach is that it promotes equity and full coverage in countries with taxpayer-funded national health services, while the downside is an increasing pressure on health services: a multi-disciplinary CRC screening team complying with the European Guidelines for Quality Assurance triggers- among other factors[8]—surveillance procedures. Furthermore, colonoscopies in the high-risk population should be performed by endoscopists with expertise in high-complexity colonoscopies.

The results of our study show that the overall volume of colonoscopies generated by the screening program is expected to double in 20 years, mainly due to the steep increase in surveillance colonoscopies. An average yearly number of 1,200 colonoscopies after a positive FIT were predicted per 100,000 inhabitants with a slight increase to 1,400 at the end of the 20-year period. Surveillance colonoscopies increased to an average of 1,000 per 100,000 inhabitants in the long term, showing certain stabilization in the last years of the 20-year simulation horizon.

Similarly, Sharp et al.[8] estimated that a FIT strategy may trigger a 33% increase in colonoscopies after 10 years, mainly due greater demand for surveillance colonoscopies. Nnoaham and Lines[21] modeled the future capacity needs in the English CRC screening program and found that colonoscopies almost doubled after 18 years, the main contributor to this increase being surveillance colonoscopies. In contrast, Rodriguez-Moranta et al.[22] suggested that endoscopic capacity in Spain can cope with widespread CRC screening with annual or biennial fecal occult blood testing, although the authors acknowledge they had stringent assumptions and that FIT was not considered. Some models aiming to inform planning of CRC screening programs propose different scenarios to buffer the pressure on colonoscopy services. For instance, during the first 3 years of implementation, there would be a shortage of endoscopy capacity that would lead to a temporary elevation of the cut-off for referral to colonoscopy in The Netherlands,[23], whereas a similar finding in Australia led to the suggestion of adding age cohorts in a stepwise fashion until all those aged 50 to 74 years were invited to screening on a biennial basis.[24]

The results of our sensitivity analysis showed that positivity is the most influential parameter in the number of colonoscopies but it should be considered together with participation and the effect of both factors over time. The effect of adherence on the number of surveillance colonoscopies was considerably lesser than those of positivity and participation.

This study has several limitations. First, the simulation of the test results was based on empirical distributions according to the results obtained from a program rather than on application of the sensitivity and specificity of tests depending on the stage in the natural history of the disease. Modeling the natural history of the disease was beyond the scope of this study. Second, surveillance colonoscopies of cancers detected within the screening program were not taken into account, as they depend on several individual factors subject to clinical decision. Third, inputs for successive screening were not stratified by age group and gender. Finally, our model does not account for the possible ‘spillover effect’ of the CRC program on general practitioners´ behavior as seen in Italy. In this regard, Parente et al.[25] described an increase in the workload of endoscopic services mainly due to an increased demand for colonoscopy in age cohorts excluded from the program.

A key strength of our work is that we used data from a CRC screening program covering around 200,000 inhabitants and that the program complied with the European Guidelines for Quality Assurance. Age and gender-specific parameters were estimated from the areas corresponding to the Colorectal Cancer Screening Program of Barcelona between 2010 and 2013 (first and second round). However, the distribution of colonoscopy results could not be stratified by age and gender because of the sample size; thus, they were held constant through the simulation horizon, stratified by initial or successive screening only.

## Conclusions

In conclusion, our model is a powerful tool for health services planning and might help to inform decision-making. Beyond the modeling/technical matters, it is our hope that this research will encourage reflection on the capacity and the demand induced by CRC screening programs, and for the need for planning of the endoscopy workforce.

## Supporting Information

S1 File Supporting Information. (DOCX)
