# Supplementary material for: Long-Term Prediction of the Demand of Colonoscopies Generated by a Population-Based Colorectal Cancer Screening Program
Source: PLoS One. 2016 Oct 12;11(10):e0164666. doi: 10.1371/journal.pone.0164666 (PMC5061389; doi:10.1371/journal.pone.0164666)
Supplement: S1 File — (DOCX) [file pone.0164666.s001.docx]

**Supporting information**

Data on the results of the screening program were obtained from the Colorectal Cancer Screening Program of Barcelona (2009-2013). The program included 31 areas of the city of Barcelona (Spain) with more than 200,000 inhabitants included in the target population. Because of geographic variations, distributions were adjusted by taking the 31 indicators of the areas as a sample and stratifying by age groups (50-54, 55-59, 60-64 and 65-69 years) and gender when substantial differences were found.

**Exclusions**

Data on the percentage of exclusions was obtained from the first round of the Colorectal Cancer Screening Program of Barcelona (2009-2011). Substantial differences were found in the percentage of exclusions among age groups and genders, and in the percentage of those exclusions due to medical reasons. Thus, a beta distribution was adjusted for the probability of exclusion by age group and gender and the percentage of exclusions due to medical reasons was applied to the sampling result of the beta distribution (see Table A1).

**Opportunistic screening**

Data on opportunistic screening was obtained from the first and second rounds of the Colorectal Cancer Screening Program of Barcelona (2009-2013). Substantial differences were found in the percentage of opportunistic screening among age groups and gender. Thus, a beta distribution was adjusted for the probability of opportunistic screening by age group and gender and the percentage of last colonoscopy between 3 and 5 years was applied to the sampling result of the beta distribution (see Table A1). Individuals sampled to have had a colonoscopy between 3 and 5 years were invited to the program 2 years later (in the next round) and the complementary percentage, which would correspond to those individuals who had had a colonoscopy in the last 3 years, were invited after 4 years (two rounds ahead).

**Table A1: Probabilistic distributions of the screening program parameters.**

|  |  | **Exclusion initial screening** | |  | **% of exclusions due to medical reasons** | |
| --- | --- | --- | --- | --- | --- | --- |
| **Age group (years)** | | **Men** | **Women** |  | **Men** | **Women** |
|  | 50-54 | 3+17*BETA(0.855,1.77) | 2+33*BETA(0.51,2.27) |  | 11.92 | 12.45 |
|  | 55-59 | 3+17*BETA(0.993,1.99) | 3+33*BETA(0.613,2.53) |  | 21.04 | 20.37 |
|  | 60-64 | 3+17*BETA(1.09,2.34) | 3+25*BETA(0.512,1.97) |  | 32.78 | 28.81 |
|  | 65-69 | 3+13*BETA(1.21,1.83) | 2+28*BETA(0.507,2.49) |  | 41.27 | 34.65 |
|  |  | **Opportunistic screening** | |  | **% to be invited after 2 years** | |
| **Age group**  **(years)** | | **Men** | **Women** |  | **Men** | **Women** |
|  | 50-54 | 0.01+3.37*BETA(2.36,3.6) | 0+3.41*BETA(2.28,3.26) |  | 40 | |
|  | 55-59 | 3.93*BETA(1.87,2.49) | 0+4.95*BETA(1.46,2.06) |  |  |  |
|  | 60-64 | 0.39+3.61*BETA(1.48,1.67) | 1+4.59*BETA(1.24,2.45) |  |  |  |
|  | 65-69 | 5.7*BETA(2.33,3.36) | 0.47+5.16*BETA(1.97,2.58) |  |  |  |
|  |  | **Participation initial screening** | |  | **Participation successive screening** | |
| **Age group**  **(years)** | | **Men** | **Women** |  | **Participants in the previous round** | **Non-participants in the previous round** |
|  | 50-54 | 18+33*BETA(2.25,1.18) | 31+26*BETA(2.38,1.85) |  | 72.75 +  17 * BETA(1.39, 0.714) | 14 +  17 * BETA(1.39, 0.714) |
|  | 55-59 | 20+35*BETA(2.46,1.4) | 36+22*BETA(1.68,1.46) |  |  |  |
|  | 60-64 | 27+31*BETA(2.07,1.27) | 35+25*BETA(2.01,1.45) |  |  |  |
|  | 65-69 | 27+29*BETA(1.55,1.02) | 35+21*BETA(1.72,1.36) |  |  |  |
|  |  | **FIT positivity initial screening** | |  | **FIT positivity successive screening** | |
| **Age group**  **(years)** | | **Men** | **Women** |  | **Men** | **Women** |
|  | 50-54 | 3+6*BETA(1.3,1.75) | 1.1+4.8*BETA(2.73,2.42) |  | 3.31 + 2.29 * BETA(2.4, 2.26) | |
|  | 55-59 | 3+11*BETA(2.63,3.54) | 1.33+6.67*BETA(3.07,2.96) |  |  |  |
|  | 60-64 | 5+12*BETA(2.49,4.1) | 2+13*BETA(2.07,4.58) |  |  |  |
|  | 65-69 | 7+11*BETA(1.39,1.86) | 2+11*BETA(2.47,4) |  |  |  |
|  |  | **Colonoscopy refusal initial screening** | |  | **Colonoscopy refusal successive screening** | |
| **Age group**  **(years)** | | **Male** | **Female** |  | **Male** | **Female** |
|  | 50-54 | 18+33*BETA(2.25,1.18) | 31+26*BETA(2.38,1.85) |  | 71 + 24 * BETA(2.24, 1.49) | |
|  | 55-59 | 20+35*BETA(2.46,1.4) | 36+22*BETA(1.68,1.46) |  |  |  |
|  | 60-64 | 27+31*BETA(2.07,1.27) | 35+25*BETA(2.01,1.45) |  |  |  |
|  | 65-69 | 27+29*BETA(1.55,1.02) | 35+21*BETA(1.72,1.36) |  |  |  |

FIT: Fecal immunochemical test.

**Participation**

Participation for initial screening was obtained from the first round of the Colorectal Cancer Screening Program of Barcelona (2009-2011). Substantial differences were found in the percentage of participation between age groups and genders. Thus, a beta distribution was adjusted for the probability of participation by age group and gender. Participation in successive screening depended on whether the individual had participated or not in the immediately previous round. Thus, consecutive participation was obtained from the second round of the same program (2011-2013), by analyzing those individuals that participated in the first round and were invited to the second round. Participation in successive screening when the individual did not participate in the immediately previous round was obtained by analyzing those individuals invited to first and second rounds who did not participate in the first round (see Table A1).

**Positivity**

FIT positivity for initial screening was obtained from the first round of the Colorectal Cancer Screening Program of Barcelona (2009-2011). Substantial differences were found in the percentage of participation between age groups and genders. Thus, a beta distribution was adjusted for the probability of a positive FIT result by age group and gender. Positivity in successive screening was obtained from the second round of the same program (2011-2013), by analyzing those individuals that participated in the first round and had a negative FIT result. See Table A1.

**Colonoscopy refusal**

Given that not all participants with a positive FIT undergo a colonoscopy due to medical reasons or refusal, the probability of not having the colonoscopy within the program was also estimated by age groups and gender for initial screening. For successive screening, one distribution was used for all participants (see Table A1).

**Colonoscopy results**

The distribution of the results of the colonoscopy after a positive FIT for initial screening were obtained from the first round of the Colorectal Cancer Screening Program of Barcelona (2009-2011). For successive screening, the results were obtained from the second round of the same program (2011-2013), by analyzing those individuals who had a negative FIT in the first round (Table A2).

**Table A2: Distribution of results from colonoscopy after a positive fecal immunochemical test at initial (n=4,253) or successive screening (n=1,874).**

|  |  | **Initial screening** | **Successive screening** |
| --- | --- | --- | --- |
|  |  | **%** | **%** |
| Negative | | 32.5 | 41.0 |
| Low-risk adenomas | | 17.4 | 20.4 |
| Intermediate-risk adenomas | | 28.5 | 26.7 |
| High-risk adenomas | | 15.6 | 8.5 |
| Cancer | | 6.0 | 3.4 |

**Colonoscopy surveillance**

The colonoscopy surveillance scheme for findings classified as intermediate- or high-risk adenomas followed the recommendations of the European Guidelines and is depicted in Figure 2.

The model assumed that surveillance colonoscopies would be carried out until the age of 79 years and that adherence would be 100% for the first surveillance colonoscopy only. For successive surveillance colonoscopies, adherence was sampled from a uniform distribution between 20% and 90% by run. Lack of adherence to a surveillance colonoscopy caused the end of follow-up.

The distribution of results after a surveillance colonoscopy was obtained from Winawer et al.,[1] considering lesions labelled as ‘pathologically advanced adenomas’ as of high or intermediate risk, the remaining adenomas as low risk, invasive cancer as is, and the rest of colonoscopies as negative (Table A3). For follow-up after the first surveillance colonoscopy, results of high or intermediate risk were considered as being of high risk in the scheme in Figure S1. For follow-up at 5 years, results of the 3-year follow-up were applied according to the risk obtained in the colonoscopy after a positive FIT.

**Table A3: Distribution of results from surveillance colonoscopy according to follow-up time and result of the colonoscopy after the positive fecal immunochemical test^a^.**

|  |  | **After high-risk adenomas** | |  | **After intermediate-risk adenomas** |
| --- | --- | --- | --- | --- | --- |
|  |  | **1 year** | **3 years** |  | **3 years** |
| Negative |  | 72.48 | 78.40 |  | 67.99 |
| Low-risk adenoma | | 24.95 | 20.41 |  | 28.27 |
| Intermediate- and high-risk adenoma | | 2.57 | 0.89 |  | 3.27 |
| Cancer |  | 0 | 0.30 |  | 0.47 |

^a^Data adapted from Winawer et al.[1]

**Validation results**

During the process of developing the model, several outcomes were systematically checked, such as the number of people invited over time (by initial and successive screenings), the participation rate, FIT positivity (by initial and successive screenings), the number of colonoscopies after a positive FIT over time (by initial and successive screenings), the distribution of colonoscopy findings after a positive FIT (by initial and successive screenings), etc. Results considered as definitive were checked by the research team by comparing them with real data from the first and second rounds of the screening program of Barcelona (Table A4). P-values for comparison between model results and real data (data not shown) were all over 0.250 except for the percentage of successive screening in the second round (p=0.007). Thus, the model was considered as valid, credible and useful for the purposes of the study.

**Table A4:** **Validation results.**

|  |  |  | **1st round** | |  | **2nd round** | |
| --- | --- | --- | --- | --- | --- | --- | --- |
|  |  |  | **Real Program** | **Model**  **(n=395)** |  | **Real Program** | **Model**  **(n=395)** |
| Participation (%) | | | 43.5% | 43.7% |  | 48.0% | 47.8% |
|  | Successive | | 0.0% | 0.0% |  | 68.3% | 62.5% |
| Opportunistic screening (%) | | | 2.1% | 1.9% |  | 1.5% | 1.2% |
| FIT positivity (%) | | | 6.2% | 6.7% |  | 5.2% | 5.3% |
| Colonoscopies after a positive FIT (%) | | | 84.1% | 83.4% |  | 84.8% | 84.5% |
|  | Negative | | 32.5% | 32.2% |  | 39.2% | 37.6% |
|  | Positive no cancer | | 61.5% | 61.6% |  | 56.2% | 57.8% |
|  |  | Low-risk adenomas | 17.4% | 17.6% |  | 19.3% | 19.1% |
|  |  | Intermediate-risk adenomas | 28.5% | 28.2% |  | 27.2% | 27.3% |
|  |  | High-risk adenomas | 15.6% | 15.9% |  | 9.7% | 11.4% |
|  | Invasive cancer | | 6.0% | 6.2% |  | 4.6% | 4.6% |
| Detection rates (per 1,000 participants) | | |  |  |  |  |  |
|  | Low-risk adenomas | | 9.0 | 9.8 |  | 8.5 | 8.5 |
|  | Intermediate-risk adenomas | | 14.9 | 15.6 |  | 12.1 | 12.3 |
|  | High-risk adenomas | | 8.1 | 8.8 |  | 4.3 | 5.1 |
|  | Invasive cancer | | 3.3 | 3.4 |  | 2.0 | 2.1 |

FIT: Fecal immunochemical test.

**Sensitivity analysis**

To simulate higher and lower participation and positivity rates, a constant factor for each run was applied to all values sampled through the distributions by age group and gender. Given an initial value p and a change δ, a decreased probability would be obtained through the formula $p*(1-\delta)$ and an increased probability using $p+\delta*(1-p)$. The factor δ was simulated using the Beta distribution with parameters α=0.4 and β=2.3 for participation and α=0.3 and β=2.4 for positivity, the latter distribution being upper bounded at 0.2.

Adherence to surveillance colonoscopies was also treated as a probabilistic parameter and was sampled from a uniform distribution between 20% and 90% for each run.

The results of the 1,750 runs were used for the sensitivity analysis. The contribution of participation, positivity and adherence to the number of colonoscopies adjusted by year was assessed using linear regression models. The response variables were: the number of colonoscopies after a positive FIT, the number of surveillance colonoscopies and the overall number of colonoscopies. Standardized coefficients (Table A5) were used to compare the importance of the factors and the unstandardized coefficients (Table A6) were used to assess the magnitude of each factor. Standardized coefficients showed that the interaction between participation and positivity had the highest importance, followed by the interactions of positivity and participation with time. Adherence had a lower impact than the rest of the parameters, although its impact increased with its interaction with time.

**Table A5: Results of sensitivity analysis: standardized coefficients of the linear regression models.**

|  |  | **Number of colonoscopies** | |
| --- | --- | --- | --- |
|  |  | **After a positive FIT** | **Surveillance^a^** |
|  |  |  |  |
| Participation | | -0.064 | -0.190 |
| Positivity | | -0.075 | -0.372 |
| Adherence | |  | -0.037 |
| Year | | -0.162 | -0.811 |
| Interactions | |  |  |
|  | Participation & Positivity | 0.935 | 0.487 |
|  | Participation & Year | 0.237 | 0.679 |
|  | Positivity & Year | 0.218 | 0.731 |
|  | Adherence & Year |  | 0.287 |
|  |  |  |  |

^a^Model adjusted without data on the first year.

FIT: Fecal immunochemical test.

**Table A6: Results of sensitivity analysis: coefficients of the linear regression models. Point estimation and 95% confidence intervals (n=1,750 runs).**

|  |  | **Number of colonoscopies** | |
| --- | --- | --- | --- |
|  |  | **After a positive FIT** | **Surveillance^a^** |
| Adjusted R-square | | 97.6% | 80.6% |
|  |  |  |  |
| Intercept | | 108,95 [98,14;119,76] | 682,84 [642,02;723,66] |
| Participation | | -1,75 [-1,95;-1,56] | -6,08 [-6,77;-5,40] |
| Positivity | | -21,75 [-23,40;-20,10] | -130,9 [-136,80;-124,99] |
| Adherence | |  | -0,88 [-1,13;-0,63] |
| Year | | -11,27 [-11,89;-10,64] | -69,55 [-72,08;-67,02] |
| Interactions | |  |  |
|  | Participation & Positivity | 4,04 [4,01;4,07] | 2,49 [2,38;2,59] |
|  | Participation & Year | 0,28 [0,27;0,28] | 0,96 [0,93;0,99] |
|  | Positivity & Year | 2,81 [2,72;2,89] | 11,41 [11,11;11,70] |
|  | Adherence & Year |  | 0,35 [0,33;0,37] |
|  |  |  |  |

^a^Model adjusted without data on year 1.

**References**

1. Winawer SJ, Zauber AG, O’Brien MJ, et al. Randomized comparison of surveillance intervals after colonoscopic removal of newly diagnosed adenomatous polyps. The National Polyp Study Workgroup. N Engl J Med 1993;328:901–6.
